# Authors’ response to comments from Nachman KE et al.

**DOI:** 10.1186/s13643-017-0611-7

**Published:** 2017-10-23

**Authors:** Annette M. O’Connor, Brent W. Auvermann, Rungano S. Dzikamunhenga, Julie M. Glanville, Julian P. T. Higgins, Shelley P. Kirychuk, Jan M. Sargeant, Sarah C. Totton, Hannah Wood, Susanna G. Von Essen

**Affiliations:** 1 Department of Veterinary Diagnostic and Production Animal Medicine, College of Veterinary Medicine, Iowa State University, Ames, IA USA; 2 Department of Biological and Agricultural Engineering, Texas A&M University, Amarillo, TX USA; 3 York Health Economics Consortium, University of York, York, UK; 4 School of Social and Community Medicine, University of Bristol, Bristol, UK; 5 Department of Medicine, Canadian Centre for Health and Safety in Agriculture, University of Saskatchewan, Saskatoon, SK Canada; 6 Department of Population Medicine, Centre for Public Health and Zoonoses, University of Guelph, Guelph, ON Canada; 7 Department of Environmental, Agricultural and Occupational Health, College of Public Health, University of Nebraska Medical Center, Omaha, NE USA; 8 College of Veterinary Medicine, Iowa State University, Ames, IA USA

**Keywords:** Systematic review, Animal Feeding Operation (AFO), Swine, Poultry, Cattle, Goat, Industrial food animal production, Community health, Bias

## Abstract

Authors’ response to comments letter to the editor from Nachman KE et al.

That our systematic review has generated debate is not surprising, as this is an important and divisive topic [1]. Nachman KE et al. [2] stated that they would have conducted the review with different eligibility criteria and risk-of-bias assessment. It is to be expected that different review teams will make slightly different judgements about the breadth of their review and the types of studies they consider appropriate to include. Our criteria were specified in advance and described in our review protocol [3]. We would welcome other groups undertaking a similar review with different eligibility criteria to investigate whether the findings are substantially different. It is important that such reviews take a systematic approach and are reported transparently, like ours, to overcome problems of narrative syntheses that have been carried out in this area in the past. We invite readers to read the Open Peer Reports of our original submission for more details of the decisions we made, including discussion about how we considered residual confounding in observational studies, the use of prevalent outcomes, chronic and acute disease, multiplicity, the age of data reported in studies, and effect measures obtained from observational studies. Below, we address specific comments made by Nachman KE et al. [2].

The authors critique our choice of risk-of-bias (ROB) tool. We selected the tool that we considered to be the most rigorous tool available for non-randomized studies (including observational designs) at the time of writing our protocol, the ROBINS-I tool [4]. The tool has been carefully developed by a large team and is based on the latest thinking in causal inference. Contrary to the impression portrayed by Nachman KE et al. [2], we modified the tool to ensure applicability to non-interventional exposures. For example, we omitted the domain that asks about adherence to interventions, we modified some elements, and we added details about exposure measurement. Nachman KE et al. [2] question our inclusion of a question about whether post-intervention variables could influence study participation. To rule out selection bias, however, it is important to determine that post-*exposure* variables did not influence study participation. We relayed our experiences with the tool to the authors of ROBINS-I so that our feedback could be incorporated into ongoing development of a tool to assess risk of bias in exposure studies [5]. We note that the draft version of this new tool for exposure studies is remarkably similar to ROBINS-I, so we do not agree with Nachman KE et al. [2] that a different approach is required in environmental health research. Nachman KE et al. [2] appear to place a higher evidentiary value on observational studies than we are willing to place. We do not believe that unavoidable limitations in observational studies lead to lower risks of bias in their findings.

Nachman KE et al. [2] also propose different eligibility criteria and questioned the exclusion of studies with only one unit of exposure. We regret that our rationale for exclusion was not clearer, so we will provide more explanation here. The concept is perhaps best illustrated using experimental design concepts. Consider a researcher interested in the comparative effects of fertilizer A and B on seed length who allocates one plant to receive A and B, and another plant to receive neither. If the researcher then measured the length of each seed produced by each plant, this would represent replication since there are multiple seeds from each plant. However, the multiple seeds would not allow stronger conclusions to be made about the effects of the fertilizers since the researcher would not be able to attribute any differences between the plants observed in the seed length to fertilizer A, fertilizer B, or other unmeasured differences such as amount of sunlight received by the plants, or random chance. Clearly, the researchers should have included multiple plants for each exposure and adjusted for the correlated outcomes from seeds within the plants. The same principle applies here, although it might be harder to detect at the macro level. For any observational study, when a single unit of exposure is used (for this topic, a single farm location compared to a single non-farm location), although the health outcome is measured on many individuals (i.e., the equivalent to the seeds), there is no replication of the exposure variable and therefore no variation in the exposure, and subsequently, no meaningful statistical analysis or causal inference. Consequently, as with the plant experiment, all of the characteristics of that unit of exposure (i.e., the farm location) will have the same association with the outcome. Further, by means of a simple but extreme example, if a study that uses a single farm location as the exposure substituted completely unrelated factors such as barn color (or owner’s surname) as the exposure variable, the data analysis would find the same association, although clearly causal inference is mistaken. To avoid this bias, we excluded studies with this characteristic.

With respect to the exclusion of the manuscript by Casey JA et al. [6], based on the approach to reporting (see Table 1), we were unable to determine whether or not people who had occupational exposure (i.e., farmers) were excluded from the study population, and therefore, we excluded the study. Other studies were more explicit that only community members were studied. As some authors of this comment are also authors of Casey JA et al. [6] (K. E. Nachman and Joan A. Casey), it is likely they have better knowledge of the study population than we were able to infer from the details reported in the publication.

**Table 1:** Text reproduced from Casey JA et al. [6]

Case Ascertainment and Control Selection Incident MRSA cases were identified primarily using laboratory cultures and secondarily by diagnosis codes (eg, International Classification of Diseases, Ninth Revision [ICD-9]) that indicated MRSA infection, as previously described.22 Cases were then classified as either CA-MRSA or HA-MRSA based on presence of health care risk factors (eg, hospitalization, surgery, dialysis, nursing home residence, indwelling device)22,31 or diagnosis more than 2 days after hospital admission using ICD-9 codes21,23,32 and Current Procedural Terminology codes. We then randomly selected patients with SSTI but no history of MRSA using 29 ICD-9 codes (eg, carbuncle, furuncle, abscess)22 and controls with no history of MRSA, and we frequency matched both groups with case patients by age (0-6, 7-18, 19-45, 46-62, 6274, ≥75 years), sex, and diagnosis or an outpatient encounter in the same year as MRSA diagnosis. The SSTI cases were evaluated as a separate case group because some SSTIs occurring during the study period were likely to have been caused by MRSA but not diagnosed as such, and high-density livestock production could cause SSTIs from other bacteria. Therefore, we selected patients with SSTIs without reference to any specific pathogen. If a control had multiple outpatient encounters during the year, a single encounter was randomly selected as the date for exposure assignment.	

Other minor issues relate to specific studies; for example, the inclusion of Avery RC et al. [7] as a study from the Community Health Effects of Industrial Hog Operations (CHEIHO) study, rather than as part of a smaller pilot investigation, is unlikely to impact our inference, but we thank the authors for pointing this out to us and to readers of *Systematic Reviews*. The authors point out that we should have been more precise when discussing the cross-sectional nature of the study by saying “many” of the studies were cross-sectional. On review of the extracted data, the longitudinal studies that were cited were designated as cohort studies and the risk of bias assessed as such [7–9].

This topic and these data are an illustration of why the scientific community should embrace the systematic review methodology and debate about the interpretation of study results. We encourage the discussion about how to assess the risk of bias in observational studies and the evidentiary value of such studies. Given the recent modification of the risk-of-bias tool for interventions for randomized studies (which are far easier to evaluate), the current tool for environmental health with the proposed name ROBINS-E may not be the final word on how to assess the risk of bias in observational studies, and open debate and discussion should be encouraged [5, 10–12].
